# Negotiating the dynamics of uncomfortable knowledge: The case of dual use and synthetic biology

**DOI:** 10.1057/biosoc.2014.32

**Published:** 2014-11-28

**Authors:** Claire Marris, Catherine Jefferson, Filippa Lentzos

**Affiliations:** a Department of Social Science, Health and Medicine, Faculty of Social Science & Public Policy, King's College London, Strand Campus, London, WC2R 2LS, UK.; catherine.jefferson@kcl.ac.uk; filippa.lentzos@kcl.ac.uk

**Keywords:** strategic ignorance, synthetic biology, dual use, biosecurity, science and technology studies (STS), innovation

## Abstract

Institutions need to ignore some knowledge in order to function. This is “uncomfortable knowledge” because it undermines the ability of those institutions to pursue their goals (Rayner, 2012). We identify three bodies of knowledge that are relevant to understandings of the dual use threat posed by synthetic biology but are excluded from related policy discussions. We demonstrate how these “unknown knowns” constitute uncomfortable knowledge because they disrupt the simplified worldview that underpins contemporary discourse on the potential misuse of synthetic biology by malign actors. We describe how these inconvenient truths have been systematically ignored and argue that this is because they are perceived as a threat by organisations involved in the promotion of synthetic biology as well as by those involved in managing biosecurity risks. This has led to a situation where concerns about the biosecurity threat posed by synthetic biology are not only exaggerated, but are, more importantly, *misplaced*. This, in turn, means that related policies are *misdirected* and unlikely to have much impact. We focus on the *dynamics* of discussions about synthetic biology and dual use to demonstrate how the same “knowns” that are denied or dismissed as “unknown knowns” in certain circumstances are sometimes mobilised as “known knowns” by the same category of actors in a different context, when this serves to sustain the goals of the individuals and institutions involved. Based on our own experience, we argue that negotiating the dynamics of uncomfortable knowledge is a difficult, but necessary, component of meaningful transdisciplinary collaborations.

## Introduction

Synthetic biology is commonly portrayed as a “dual use” field of science that increases the risk of attacks using bioweapons, especially from terrorists operating outside state organisations. In a previous paper, we described and challenged five “myths” that underpin this dominant discourse, and showed how it is based on misleading assumptions about bioweapons and bioterrorists, and about the meaning of synthetic biology's aim to “make biology easier to engineer” (Jefferson *et al*, 2014a). In this article, we demonstrate how this leads to a situation where these concerns are not only exaggerated, but are, more importantly, *misplaced*; and how this, in turn, leads to misdirected policies that have a limited impact on the reduction of biosecurity risks. We draw on Steve Rayner's concept of “unknown knowns” to investigate the way in which assumptions about the dual use of synthetic biology persist, despite these seemingly undesirable consequences and the availability of knowledge that would undermine them.

Rayner (2012, p. 108) states that: “in drawing attention to what we know we know, what we know we don't know and what we don't know we don't know, Rumsfeld altogether omitted what is possibly the most intriguing combination: what we don't know we know”. Rayner therefore adds a fourth category to the three coined by former US Secretary of Defense Ronald Rumsfeld (“known knowns”, “known unknowns” and “unknown unknowns”). These are “unknown knowns”: “knowledge that exists somewhere else in society but is not known here” (ibid., p. 108). Rayner treats ignorance “as a necessary social achievement rather than as a simple background failure to acquire, store and retrieve knowledge” (p. 108). In some cases, this is because knowledge is deliberately concealed by those who hold it, in order to avoid individual or organizational responsibility or embarrassment” (p. 108). A number of cases are explored in the other papers published alongside Rayner's in a special issue of *Economy & Society* on “strategic ignorance” (McGoey, 2012),^1^ but Rayner's focus is different, because he uses the term “strategy” “to include not only deliberate attempts to manage information, but also implicit or even completely unconscious strategies” (op cit. p. 113) and situations where there are “strong social pressures to forget the inconvenient truths” (p. 109). He thus explores “unknown knowns of a particular sort: those which societies or institutions actively exclude because they threaten to undermine key organizational arrangements or the ability of institutions to pursue their goals” (p. 108). From this perspective, “the social construction of ignorance is not only inevitable, but actually necessary for organizations, even entire societies, to function at all” (p. 122). This is because (p. 107):

To make sense of the complexity of the world so that they can act, individuals and institutions need to develop simplified, self-consistent versions of that world. The process of doing so means that much of what is known about the world needs to be excluded from those versions, and in particular that knowledge which is in tension or outright contradiction with those versions must be expunged. This is “uncomfortable knowledge”.

Unknown knowns therefore remain unknown because they constitute “uncomfortable knowledge” which is “disruptive knowledge” (p. 113).

We argue that the current dominant portrayal of the potential dual use of synthetic biology can be seen to illustrate such a simplified and self-consistent worldview, and identify three unknown knowns that are relevant to understandings of the dual use threat posed by synthetic biology but have been excluded from policy discussions. We describe how these “inconvenient truths” have been systematically ignored and argue that this is because they are perceived, by organisations involved in the promotion of synthetic biology as well as by those involved in managing biosecurity risks, as a threat to their ability to pursue their goals and justify their existence.

Rayner describes four strategies employed by organisations to manage uncomfortable knowledge: denial, dismissal, diversion and displacement. We observed such strategies in our fieldwork, but suggest that Rayner's framework underestimates the extent to which knowledge and ignorance can be differentially constructed over time, and also across different arenas. We therefore focus on the *dynamics* of discussions about synthetic biology and dual use and describe situations where the same knowns that are denied or dismissed as unknown knowns in certain circumstances are then mobilised as known knowns by the same category of actors in a different context. This analysis reveals how, in some circumstances, key concepts such as “tacit knowledge”, “de-skilling” and “engineering” are *re-interpreted* in order to sustain the goals of the biosecurity and synthetic biology institutions involved.

## Methods

The research presented here is based on participant observation in scientific and policy arenas related to biosecurity and to synthetic biology. Filippa Lentzos and Catherine Jefferson have been participating in a wide range of events on biosecurity, bioweapons, arms control and non-proliferation for over a decade. Claire Marris and Filippa Lentzos have been participating in a wide range of scientific and policy initiatives on synthetic biology for the last 5 years, and Catherine Jefferson for the last 2 years. This has included scientific meetings ranging from large-scale international conferences such as those in the SBx.0 series, national conferences and workshops, and laboratory meetings at the Centre for Synthetic Biology and Innovation (CSynBI) that all three authors are members of, as well as informal conversations with synthetic biology researchers at CSynBI and elsewhere. In the field of biosecurity, this has included meetings of the Biological Weapons Convention (BWC), the Pugwash study group on the BWC, and meetings organised by the Foreign and Commonwealth Office, Wilton Park, Chatham House, National Academies of Science, Royal Society, World Health Organisation and others.^2^

The insights reported in this article emerged from this immersion in the worlds of synthetic biology and biosecurity, which provided the authors with regular opportunities to interact with synthetic biologists, government officials, security analysts, diplomats, public health officials, law enforcement agents, DIY biologists and others who have assembled around the problem of the dual use of synthetic biology. These interactions took place in natural settings (as opposed to, for example, an interview setting), in places and during events that these actors – and the authors – were participating in through the course of their work.

It is through this fieldwork that we became aware of the prevalence of particular ways of framing the issues at stake, and were able to analyse how actors mobilised particular arguments. We also carried out a review of academic literature from both social and natural sciences, and grey literature from policy institutions. We then organised a workshop with key UK stakeholders (scientists, policy experts, science journalists and social scientists with professional roles related to synthetic biology and/or biosecurity) to discuss our preliminary findings (Jefferson *et al*, 2014b).

## Framing Synthetic Biology and Dual Use

### Dominant framing of dual use research

In the context of security policy, dual use refers to the concern that “legitimate” science and technology has the potential to be “misused” for the development of prohibited weapons. In recent years, the focus of dual use concerns has shifted from “technologies” to “research”. This is illustrated by the influential 2004 policy report produced by the US National Research Council (NRC) (2004), *Biotechnology Research in an Age of Terrorism: Confronting the Dual Use Dilemma*, which viewed the inherent characteristics of scientific research as a dual use concern. This marked a departure from earlier dual use policies aimed at preventing the spread of potentially dual use technologies or material through, for example, export controls or legal obligations placed on researchers working with select agents (McLeish, 2007). The prevailing framing of dual use in contemporary discussions is typified here by Miller and Selgelid (2007, p. 523):

The dual-use dilemma arises in the context of research in the biological and other sciences as a consequence of the fact that one and the same piece of scientific research sometimes has the potential to be used for bad as well as good purposes. [...] It is an ethical dilemma for the researcher because of the potential actions of others, e.g., malevolent non researchers who might steal dangerous biological agents, or make use of the original researcher's work.

This framing of dual use implies that a “piece” of research intended for “good purposes” could be readily and directly misapplied for “bad purposes” with little to no consideration of the challenges involved. Furthermore, dual use is framed as an “ethical dilemma” and thus one that is a quandary “for the researcher” who must protect their research from the possible misuse of “others”, who are “non researchers” (that is, malevolent outsiders). As we shall see, this particular framing of dual use concerns resonated with the central promise of synthetic biology to “make biology easier to engineer”, and led to the entanglement of these two areas.

### Unknown knowns about dual use and synthetic biology

We have previously highlighted five “myths” associated with the dominant discourse about the potential dual use of synthetic biology for malevolent purposes (see Box 1) and argued that these are based on simplistic understandings of synthetic biology's ability to “engineer biology”, of bioweapons and of the intentions of bioterrorists. We demonstrated how each of these myths are contradicted, or at least complicated, by empirical evidence about the real-life practices of professional synthetic biologists, iGEM teams, DIY biologists and would-be bioterrorists (Jefferson *et al*, 2014a).

We acknowledged that each of the myths contained some elements of truth, and did not seek to make a simplistic contrast between myths and reality. Instead, we used the term myth to indicate that the assumptions behind each of these myths were portrayed as so self-evident in policy and scientific arenas that they did not need to be supported by specific evidence. As a result, the dominant narrative that is based on these myths circulates unchallenged and becomes a story that is told and retold, and becomes further crystallised through each retelling. Marris (2001) used the term “myth” in a similar way with respect to assumptions about public views on genetically modified crops to underscore “that they appear so ‘evident' that no further substantiation seems to be needed” (p. 545).

Here, we identify three key bodies of knowledge that constitute foundational insights from the fields of STS and innovation studies that challenge these myths. These are considered obvious in STS circles but are ignored in discussions about the dual use of synthetic biology in policy arenas and public debates. They therefore constitute unknown knowns.

#### First unknown known: The importance of tacit knowledge

First, there is a set of findings from the sociology and philosophy of science about the role of tacit knowledge for the success of scientific projects. Tacit knowledge refers to skills, knowledge and techniques that cannot be readily codified and are obtained through experience, by working in teams, and by participating in professional scientific networks through a process of “learning by doing” or “learning by example”. Such local, specialized skills and know-how cannot be readily transferred or obtained simply from reading scientific journal articles, yet this type of knowledge is crucial to success in scientific practice (Polanyi, 1966; Collins, 2007, 2010).

The importance of tacit knowledge is frequently overlooked in the dominant narrative of the dual use threat associated with advanced biosciences, where the focus tends to be on access to biological materials and published research, rather than on human practices and institutional dimensions. We argue, together with Vogel and Ben Ouagrham-Gormley (Ben Ouagrham-Gormley and Vogel, 2010; Ben Ouagrham-Gormley, 2012; Vogel, 2013), as well as Revill and Jefferson (2013), that recognising the importance of tacit knowledge is crucial for more sophisticated assessments of biosecurity threats. Yet this remains an unknown known. We believe that this is because acknowledging the importance of tacit knowledge in all scientific endeavours could be perceived as undermining claims made about the revolutionary nature of synthetic biology, based on its ability to “black box” complex knowledge and to reduce the need for specialised craft skills. It would also challenge claims made about the reproducibility of scientific experiments and the ability of synthetic biology to improve this.

#### Second unknown known: Challenges to the linear model of innovation

Second, there is a relevant body of knowledge about the relationship between science, technology and innovation, derived from innovation studies. McLeish and Nightingale demonstrate how the current framing of dual use is premised on a “linear model” of “technology transfer” that is seriously challenged by social science scholarship in this area (McLeish, 2007; McLeish and Nightingale, 2007). This body of knowledge goes beyond the issue of tacit knowledge and points to the need for the alignment of particular organisational structures, processes and practices in the production of technology. Vogel and Ben Ouagrham-Gormley have documented in-depth how these factors have constrained bioweapons programmes in the United States, Soviet Union and Iraq (Ben Ouagrham-Gormley, 2012; Vogel, 2013; Ben Ouagrham-Gormley, 2014). Specific local organisational structures and divisions of labour have to be developed for every stage of the bioweapons process, from research, to development, to small-scale production, large-scale production, testing and weaponisation. This is not a linear process that moves smoothly from one stage to the next: each stage requires the expertise of different people in different teams, each cooperating together. This crucial set of insights has not been picked up in either the policy or academic discussions in this area. For example, even though challenging the understanding of dual used based on a linear model of innovation was central to the 2007 article by McLeish and Nightingale, none of the 21 citations to this article mention this argument. We believe that it remains an unknown known in discussions about dual use research because it implies that “science is far less likely to take centre stage” in innovation processes (McLeish and Nightingale, 2007, p. 1643). This means that it consitutes uncomfortable knowledge for insitutions conducting or promoting scientific research.

#### Third unknown known: Dangerousness depends on context

Scholars in the field of science and technology studies (STS) have questioned consequentialist use/abuse framings of technology. Rappert (2005) has shown how “attempts to devise prohibitions [of weapons] regularly entail delineating certain actions or artefacts as inappropriate, unacceptable, and so forth” (p. 212) and how this requires “cutting up complex socio-technical assemblages” (p. 227). In disputes about the use of weaponry, participants attempt to distinguish “social” and “technical” factors in order to identify the principal source for concern. The role of technology in contributing to inappropriate acts is commonly framed “in terms of the use of neutral tools that can be employed for good or bad purposes, depending on the user” (p. 213).

**Figure 1:**
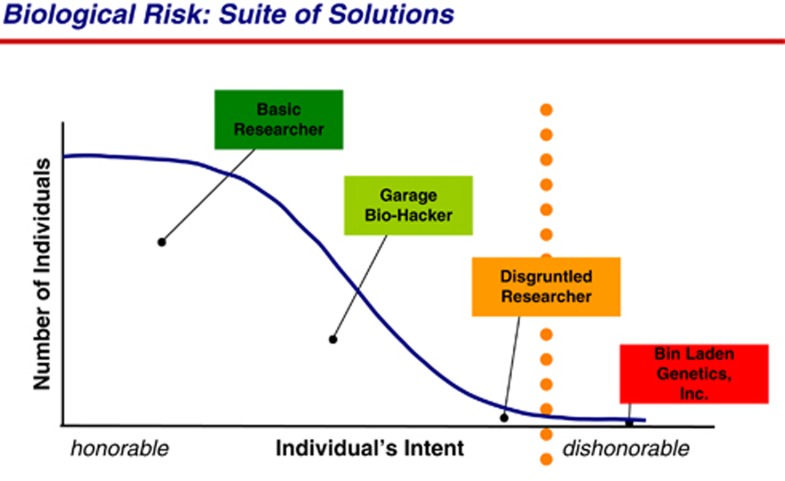
Classifying honourable/dishonourable intent. *Source*: “2003 Synthetic Biology Study” (p. 16) by Drew Endy, licensed under a Creative Commons Attribution-NoDerivs 3.0 Unported (CC BY-ND 3.0). Available from: hdl.handle.net/1721.1/38455

**Figure 2:**
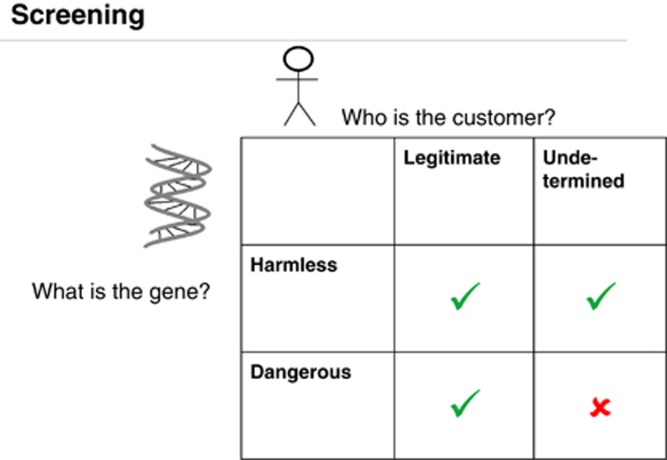
Gene and customer screening as advocated by the International Association Synthetic Biology. *Source*: Powerpoint presentation on “Synthetic Biology: Addressing Global Security” by Markus Fischer, representing the International Association Synthetic Biology (reproduced with the kind permission of Markus Fischer).

**Figure 3:**
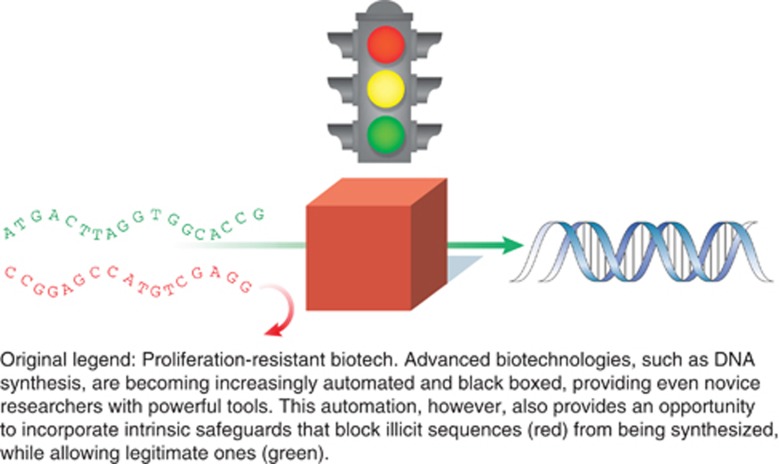
Traffic light portrayal of DNA sequence screening. Reprinted by permission from Macmillan Publishers, Ltd: *Nature Biotechnology*, Nouri, A. and Chyba, C. F., “Proliferation-resistant biotechnology: An approach to improve biological security” 27(3): pp. 234–236, p. 235, © 2009.

Drawing from Bruno Latour's analysis of the National Rifle Association's slogan “Guns don't kill people, people do”, Rappert points out that, “asking whether it is the user or the gun that is the ‘real' agent missed how the weapon and the person are hybridised together in a locally accomplished assemblage” (ibid., p. 215). Rappert shows how, in practice, it is very difficult to distinguish acceptable from unacceptable uses of military technologies. Evaluating the acceptability of uses of scientific research, knowledge and materials is even more difficult. Distinguishing between the knowledge and techniques necessary for defense and those required for the production of weapons is not straightforward and only becomes evident in some wider context. Balmer (2006) has also pointed out that, in the context of security “[k]nowledge is constructed as dangerous within particular spaces” (p. 715). Yet, as we discuss below (and illustrated by Figures 1, 2, 3), dominant narratives and policy initiatives in this area largely assume that establishing such binary categories without any reference to context is a relatively simple matter. Acknowledging this third unknown known would entail admitting that policies based on such simplified categories are unlikely to be effective. This, we argue, is why this knowledge is uncomfortable for some actors and thus remains unknown in certain arenas.

#### Absence of these three bodies of knowledge in the literature

We have conducted a review of contemporary (since 1999) academic and non-academic literature about synthetic biology and/or biosecurity, searching, specifically, for any instances in which a less simplified and linear version of dual use, and of the relationship between science and technology, was portrayed. The corpus included influential reports such as: the National Research Council (NRC) (2004) report, *Biotechnology Research in an Age of Terrorism* (more than 300 citations in Google Scholar^3^); the US Presidential Commission for the Study of Bioethical Issues (PCSBI) (2010a) report, *New Directions: The Ethics of Synthetic Biology and Emerging Technologies* (150 citations); the European Group on Ethics's (2009) *Opinion no. 25* – *Ethics of Synthetic Biology*; the report commissioned by the J. Craig Venter Institute (JCVI), *Synthetic Genomics: Options for Governance* (Garfinkel *et al*, 2007) (90 citations); the UK Royal Academy of Engineering (2009) report *Synthetic Biology: Scope, applications and implications* (60 citations); and the US National Science Advisory Board for Biosecurity (NSABB) (2010) report *Addressing Biosecurity Concerns Related to Synthetic Biology*.

The overwhelming result from our analysis is that both the grey literature from policy institutions and the academic literature in this area does not draw upon, or even acknowledge, the three bodies of knowledge identified above (except of course for publications by the STS authors who developed this knowledge and that we previously cited). Demonstrating the evidence of the absence of a phenomenon is always harder than demonstrating its presence. As an illustration, the term “tacit knowledge” did not appear in any of the reports listed above. Instead, both these sets of literature replicate the 5 myths we previously characterised. We did find a small number of exceptions where tacit knowledge was discussed, but noticed that in these cases the term itself (and the related STS literature) was not utilised, and the term was redefined in significant ways.

#### Tacit knowledge as discussed by policy analysts

One of the very few examples we found of a mention of “tacit knowledge” in our literature review was an article by Mukunda *et al* (2009, p. 12). Here, knowledge is recognised as raising barriers that currently hinder “even the most skilled bioengineers”, but this recognition is mobilised in the context of an argument about how synthetic biology will in due course “eliminate” these barriers, because it seeks to “make biology easier to engineer”, and it is assumed that this will make it possible “for *everyone* to engage in successful bioengineering”:^4^

SynBERC Director Jay Keasling described the high demands for tacit knowledge that currently hinder even the most skilled bioengineers and said that SynBERC's vision is to “make biology easier to engineer.” The emphasis here is crucial. SynBERC certainly seeks to produce various specific applications but that is not its primary goal. Instead the center seeks to eliminate the barriers, particularly those involving tacit knowledge, that make it more difficult for *everyone* to engage in successful bioengineering. […] Synthetic biology includes, as a principal part of its agenda, a sustained, well-funded assault on the necessity of tacit knowledge in bioengineering and thus on one of the most important current barriers to the production of biological weapons. (Emphasis in original)

A rare example of an in-depth discussion of tacit knowledge occurs in a 2011 article by Tucker, a prominent expert on chemical and biological weapons (Tucker, 2011). However, Tucker did not mention the concept at all in his far more influential 2006 article (Tucker and Zilinskas, 2006). Moreover, the 2006 article has become a touchstone in the field, and has been cited 96 times, whereas the 2011 article has only been cited five times.^5^ This illustrates how knowledge about the importance of tacit knowledge tends to be ignored in policy circles, and thus constitutes an unknown known.^6^

#### Reframing of tacit knowledge at the BWC

In this background paper prepared by the BWC Secretariat, tacit knowledge is portrayed as something that can be “transferred” through the availability of web-based technologies (Implementation Support Unit, 2011):

Although first class research continues to rely heavily upon tacit knowledge, the availability of web-based technologies is facilitating the transfer of tacit knowledge through the creation of worldwide formal or informal learning communities or partnerships […] an area for future in-depth analysis is the changing nature of tacit knowledge, of which intangible technology is a subset, as kits and other resources make it easier for less skilled individuals to carry out work that once required significant training.

While some aspects of weak tacit knowledge can be rendered more explicit under certain circumstances (Collins, 2007, 2010; Revill and Jefferson, 2014), the assertion that “kits” and other resources will make it easier for less-skilled individuals to conduct sophisticated biological research fails to recognise stronger forms of tacit knowledge such as the skills, mechanical techniques and idiosyncratic know-how obtained by individuals through trial-and-error problem-solving or through a master–apprentice style relationship.

The issue of tacit knowledge was recently raised again at the BWC (Implementation Support Unit, 2014, p. 3):

An eighth trend may now be added: the growing tacit knowledge requirement for life science work. Researchers attempting to replicate experiments raised the alarm on the growing difficulty of reproducing research; this issue has become so severe that those seeking to replicate results obtained at another lab are now encouraged to do so through joint work. This trend is, in part, driving the seventh trend: research collaborations are set up to bring together the “barrage of high-end equipment that no one can afford,” but also to pool the tacit knowledge required to effectively employ these pieces of sensitive equipment.

The way in which tacit knowledge is framed in this instance bears little relation to the understanding developed by Revill and Jefferson (2014) in the context of biosecurity. In this framing, tacit knowledge is seen as a new, growing – and “alarming” – “trend”, not an ordinary component of all knowledge production in scientific practice. This is particularly striking given that this paragraph specifically cites the article by Revill and Jefferson (and our workshop report) as a source.

#### Studies of would-be terrorists that demonstrate real-world context

Our literature review revealed that whenever studies are conducted about the real-life activities of would-be bioterrorists, the difficulties encountered by these groups are highlighted. Like the studies conducted by Ben Ouagrham-Gormley and Vogel about state-sponsored bioweapons programmes, these studies provide data from the real world which challenges assumptions made in the reified versions of the world that dominate policy arenas. For example, when Danzig *et al* (2011) documented the achievements – or more accurately the failures – of the Aum Shinrikyo cult's attempts to develop biological weapons, they suggested that “[t]he distinction between explicit (book) knowledge and tacit (hands-on) knowledge may be helpful here” to understand the difficulties faced when developing biological weapons as opposed to chemical weapons (p. 35). Tacit knowledge is understood here as something that is particularly relevant to some areas of science (biology), but not others (chemistry): “[d]eveloping biological weapons appears to require more tacit knowledge, while chemists may be adequately positioned to develop weapons after consulting relevant documentation” (p. 35). However, Polanyi (1966) considered that tacit knowledge applied to all aspect of scientific research and Collins has argued that it even applies to off-the-shelf consumer appliances supplied with instruction manuals, such as bread machines (Ribeiro and Collins, 2007).

In other studies of the activities of would-be terrorists, the authors do not couch their findings in terms of tacit knowledge but also point to the real-life challenges involved (Leitenberg, 1999; Kaplan, 2000; Wheelis and Sugishima, 2006). Leitenberg noted that (p. 156): “The experience of the Aum is therefore in marked contrast to the legion of statements by senior US government officials and other spokesmen claiming that the preparation of biological agents and weapons could be carried out in ‘kitchens', ‘bathrooms', ‘garages', ‘home breweries', and is a matter of relative ease and simplicity”. Moreover, Leitenberg pointed out that “the serial propagation of misinformation” about the Aum's capacity to produce lethal biological weapons continued despite the fact that classified US government evaluations of the Aum was similar to his own, and lamented the fact that US biosecurity policy was launched “on the basis of this and even greater errors” (p. 157). In the next section, we explore the impact that the failure to acknowledge unknown knowns has had on policy discussions and initiatives related to the threat posed by the dual use of synthetic biology.

## Misdirected Policies

As argued by Rayner, the social construction of ignorance is necessary for organisations to function. However, in some cases, it can lead to situations “where the accepted version [of the world] excludes knowledge that is crucial for making sense of and addressing the problem” (op cit., p. 107). We suggest that the current accepted framing of dual use of synthetic biology is an example of such a “dysfunctional” case (p. 122). The structured construction of ignorance by biosecurity and synthetic biology institutions directs the policy gaze towards certain issues, and away from actors, institutions, problems and solutions that come to the fore when different versions of the world are taken into account (see Table 1). Policy discussions about the dual use threat of synthetic biology tend to focus on: (i) the control of access to materials; (ii) identifying malevolent users and keeping them out; (iii) assessing “pieces” of research; and (iv) programmes to educate scientists and make them behave more responsibly.

### Control of Access to Materials

Policy discussions focus on access to materials such as synthetic DNA, machines and materials to synthesise DNA, and online access to DNA sequences. This is evident when one considers that screening of orders by DNA synthesis firms is the only policy measure that has (to some extent) been implemented to respond to concerns raised about the potential misuse of synthetic biology. In 2008–2009, two separate consortia of DNA synthesis companies (the International Gene Synthesis Consortium, IGSC and the Industry Association Synthetic Biology, IASB) developed competing protocols specifying what companies should do to screen customer orders for possible biosecurity threats; and these have become the basis for self-regulation by these firms. In 2010 the US government independently issued its own guidance, but its recommendations were weaker and narrower than those developed by the companies (Maurer, 2012).

One of the earliest reports about the governance of synthetic biology is the 2007 report commissioned by the JCVI (Garfinkel *et al*, 2007) (cited 92 times). Virtually all of the “options for governance” discussed in this report focus on access to synthetic DNA. Others focus on broadening the review responsibilities of Institutional Biosafety Committees and/or establishing a National Advisory Group in order to identify and evaluate “risky experiments” that might generate “dangerous knowledge”, which relates to the third policy focus we have identified. One option suggests compiling a biosafety manual for synthetic biology laboratories, and the remaining options focuses on education programmes “about risks and best practices” for scientists, which relates to the fourth policy focus we have identified.

The PCSBI also focused on this issue during its 2010 auditions about the ethics of synthetic biology. In his deposition, Ralf Wagner, Chief Executive Officer of the DNA synthesis company GeneArt, which was part of the IGSC, described his company's screening strategy and implied that the process for judging gene sequences is relatively straightforward (PCSBI, 2010b, Session 7):

there is highly sophisticated software tools already available amongst the gene synthesis companies where sequences on a protein level, on a sequence level are very, very carefully screened, and we receive hits. The hits are compared to black lists, to white lists, and it's not just an expert software. It also has a user friendly interface service with very clear cut results so that you're pretty sure that, based on the standards we have set, that we are able to appropriately judge the sequences that we get

He also stressed that “We do not like to work in gray zones, so we would like to have very, very clear cut criteria for screening”. Figure 2, which represents the IASB screening strategy, provides a similarly clear-cut binary portrayal of the screening process; and the traffic light portrayal of screening in Figure 3 suggests that is possible to automatically distinguish “illicit” and “legitimate” DNA sequences.

In the Q&A session that followed Wagner's talk, however, concerns were raised about how these protocols were implemented in practice, and a different picture emerged, with Wagner admitting that there were indeed “gray zones” (PCSBI, 2010b, Session 7):

there will be, there will stay, a certain gray zone for those genes which are not precisely described regarding their pathogenic potential. […] And really to make a clear statement for each individual gene […] in a given new context, I believe this is extremely, extremely demanding and difficult. And, today, I do not have a clear answer.

This illustrates how the simplified, binary version of the world that underpins the screening policies advocated by the IGSC and IASB falls apart when real-world considerations are introduced. The disagreement between the companies behind these two consortia centred on whether human screeners could be replaced by a pre-defined list of dangerous sequences. List-based systems facilitate automation (as portrayed in Figure 3) and cost almost nothing to implement, but are less effective because “current lists are notoriously incomplete” (Maurer, 2012, p. 7). Taking Rappert's insights seriously would, however, suggest that such lists can never be complete and that a list-based system can never be fully effective because it requires “cutting up complex socio-technical assemblages”. These screening protocols seek to assess the dangerousness of actors and materials in isolation from the context of use, and this is, in practice, very difficult if not impossible. This is further illustrated by the fact that “obvious next steps” have not been implemented, such as merging the IASB and IGSC protocols into a single standard or completing an open source archive where companies can share data and judgments about which DNA sequences constitute threats Maurer (2012, p. 8). Indeed, in recent years both the IGCS and the IASB have been inactive and biosecurity concerns do not feature prominently on the Websites of DNA synthesis companies such as DNA2.0, GeneArt and Integrated DNA Technologies who initially took a lead in this area.

### Keeping outsiders out

The common framing of dual use tends to assume that malevolent actors operate only outside government, university and company laboratories, which are implicitly portrayed as necessarily “legitimate” institutions. The focus is not so much on the potential use of synthetic biology to develop bioweapons but on the (mis)use of synthetic biology for *bioterrorism* (e.g. Bernier and Rose, 2014; Garfinkel *et al*, 2007, p. 5; Royal Academy of Engineering, 2009, p. 43; PCSBI, 2010a, p. 72). As illustrated by the quote from Miller and Selgelid above, the central idea is that “malevolent non researchers” “might steal” dangerous materials and knowledge from benevolent researchers. This leads to policy initiatives that focus on keeping these outsiders out, by limiting access to knowledge and materials (which is assumed to be neutral) to legitimate insiders. This framing is common to all discussions on dual use, but, in the context of synthetic biology, this phenomenon has been amplified by the way in which leading synthetic biologists have deployed particular notions of “de-skilling” and “the engineering of biology”, and made the associated claim that “everyone” can (or soon will be able to) do it.

In our fieldwork, we have been struck by the way in which discussions of the risks associated with synthetic biology routinely drift to focus on outsiders, and how DIY “biohackers” have come to epitomise this category (e.g. Royal Academy of Engineering, 2009, p. 43). DIY biology is routinely raised as an issue of concern and inordinate amounts of time are devoted to discussing the (presumed) activities of these (presumed) amateurs.^7^

As illustrated in Figure 2, the screening procedures for synthetic DNA orders assume that “legitimate” customers can be identified in a straightforward way. In Figure 1, we also see a binary ordering of individuals according to their “honourable” or “dishonourable” intent.

In his deposition to the PCSBI, Wagner identified the need for “an internationally harmonized list of suspicious persons and organizations” but admitted that “this is a topic that needs maybe more deeper discussion”. Methods for customer screening are far less advanced than those for sequence screening and mostly rely on the fact that the client's postal address is not a residential address or a PO box, and ensuring that the address owner is a legitimate organisation.

Yet, the post-9/11 “Amerithrax” attacks, where five anonymous letters containing a deadly strain of anthrax were sent to media outlets and the US Senate, which is one of the few examples available of an actual bioterrorist attack, have been attributed to Bruce Ivins, a professional biologist working for the Department of Defense. This is a “legitimate” organisation that would not generate a “hit” in such screening procedures. One way to recognise this historical fact without disrupting the binary worldview is to portray Ivins as an outsider. Thus, Flower (2014) depicts him as a “rogue scientist” who is a “trained scientist possessing expert skills and with access to pathogens, reagents and cutting-edge technology” and who “might appear outwardly to be part of the conventional academic establishment except that their motives impel them to act covertly outside the accepted ethical and legal boundaries of their profession”. We see here how the boundaries between insiders and outsiders become more complex, and can be shifted to accommodate uncomfortable knowledge.

Following the identification of Ivins as the perpetrator of the anthrax letters, there has been some increased focus on personnel reliability programmes to “vet” people permitted to access high-containment laboratories and select agents using, for example, security checks and psychological testing. However, the value of personnel reliability testing has been called into question, particularly given that Ivins was himself subject to an evaluation (Royal Society, 2009). Moreover, this approach implies that it is possible to delineate complex behaviours such as reliability and trustworthiness to identify “rogue scientists”.

The fact that debates on the dual use of synthetic biology focus on its ability to reduce barriers to entry deflects attention from insiders. Much less attention is paid to research sponsored by large powerful states and conducted in professional well-funded laboratories that are generally regarded as legitimate: work conducted in secret in military labs, or funded by defence agencies in academic and commercial institutions. Military sources of funding have been significant for synthetic biology. Funding from the Office of Naval Research helped to lay the foundation for the field in the 1990s (Check Hayden, 2011) and the US Defense Advanced Research Projects Agency (DARPA) has now become one of the largest sources of US federal funding for synthetic biology. DARPA spent $35 Million (M) on its “Living Foundries” programme in 2012–2014, and plans to spend a further $115M in 2014–2018 (Jackson, 2013). In August 2014, DARPA announced a further $42.5M for its “Biological Robustness in Complex Settings Programme” (DARPA, 2014). Military funding for synthetic biology has also been significant in the UK. In 2012, multiple agencies including the Defence Science and Technology Laboratory (DSTL) invested £2.4M in the “Joint Synthetic Biology Initiative”, and in 2014 the DSTL and the Centre for Defence Enterprise announced a further £1M for a research programme on “Synthetic Biology Applications in Defence” and a further £3M (potentially) in phase 2 of this programme. Although one news item in *Nature* reported that “bioengineers debate use of military money”, we have found that this issue is not often raised as a concern in discussions among synthetic biologists, and is not discussed in the literature on the potential dual use of synthetic biology. Moreover, the policies that have been discussed or implemented do not focus on these insiders and sometimes explicitly exclude them from consideration. Thus, Church's (2004) *Non-proliferation Proposal* naturally assumes that “there will be government agencies which are exempt from the entire system”. In order to defend their integrity, some of the synthetic biologists cited by Check Haden stressed the potential beneficial outcomes of military-funded research, such as “greener” explosives or environmental sensors to detect mines. Indeed, an Editorial in *Nature* focused entirely on “dual use benefits” of military research – referring here to dual use as the civilian benefits derived from research funded by military institutions.

### Reviewing specific “pieces” of research

Conceptions of biological threats have broadened over the last decade beyond dual use technologies and specific lists of pathogens to encompass a focus on scientific research. In this context, the notion of “dual use research of concern” (DURC) has gained increasing traction in the policy community and, in the case of the United States, has led to policy action. For example, in 2004 the NSABB was established in order to “provide advice on and recommend specific strategies for the efficient and effective oversight of federally conducted or supported dual use biological research” (DHHS, 2014). In 2012, the US Government issued the *Policy for Oversight of Life Sciences Dual Use Research of Concern*, which requires US federal departments and agencies that fund life science research to “identify and manage the risks associated with dual use research of concern” (DHHS, 2014).

Within debates about DURC, discussions focus on whether it is appropriate to limit the freedom of scientists to conduct research that is deemed to be inherently “dangerous” if “transferred” to malevolent outsiders, or to restrict publication of the results from that research. This emphasis on the dual use threat associated with discrete “pieces” of research fails to take into account the complexity of innovation processes and the intangible barriers that would need to be overcome in order to exploit any single “piece” of research for the purposes of bioweapons development (second unknown known). Moreover, a focus on published, codified knowledge fails to take into account the importance of tacit, local and collective knowledge for successfully replicating scientific results (first unknown known). The focus on DURC and the threat of deliberate release of lethal pathogens also diverts attention away from more pressing considerations about biosafety and the risk of *unintended* release of pathogens.

Rappert (2014) examines the limitations of this risk-benefit framing of experiments of concern, and highlights that benefits are as challenging to anticipate as risks, and both could be subject to different interpretations. Interestingly, the idea that “pieces of research” can be assessed as inherently dangerous or not contradicts the routine claim that science is neutral and only becomes value-laden when used by specific actors. Indeed this belief in neutral science underpins policies aimed at controlling access to knowledge and those aimed at keeping outsiders out. These two contradictory claims sit alongside each other in the dominant narrative on dual use, with either one stressed in different contexts.

### Programmes to educate scientists and make them behave more responsibly

Interest in “dual use education” for life scientists has grown considerably in the past decade. There have been calls for action in this area at the state, regional and international levels, including the G8 “Global Partnership against the spread of weapons and materials of mass destruction”, the European Union and the BWC. When, in 2011, BWC Member States agreed to annually review developments in science and technology related to the treaty, they specifically highlighted education and awareness-raising about the risks and benefits of life sciences and biotechnology (BWC, 2012, p.23).

Research conducted in diverse countries (for example, the United Kingdom, the United States, Australia, Argentina, Israel, Kenya, Uganda and Ukraine) has shown that practicing life scientists have little awareness of the dual use threats associated with their research, or of the BWC; and this has led to calls for the incorporation of dual use education in university life science courses (Dando, 2011; Espona and Dando, 2011). Proponents argue that dual use education is necessary to ensure that life scientists are aware of misuse risks, and that early engagement provides the foundations for a more informed discussion between the science and security communities. However, echoing the “ethical dilemma” noted earlier, emphasis is also placed on education as a means to foster a culture of responsibility within the life science community so that the scientists might protect their work from “malign misuse” (Dando, 2011). As a result, there is considerable focus on the need to “educate” scientists about “ethics” and to make them “aware” of the potential misuse of their research by “hostile” actors. This framing is evident, for example, in the articles published in a special issue of *Medicine, Conflict and Survival* entitled “Preventing the hostile use of the life sciences and biotechnologies: Fostering a culture of biosecurity and dual use awareness” (Dando, 2012), which includes one article devoted to synthetic biology (Edwards and Kelle, 2012).

While education and awareness-raising initiatives are a worthwhile governance measure within a larger strategy to address misuse concerns in the life sciences (Lentzos, 2008), the way in which they are framed fails to take into account the unknown knowns identified in this article. This means that, like the other policies discussed above, they are underpinned by a binary understanding, where benign use can be clearly distinguished from “malign misuse”, and “exceptionally dangerous research and publication” identified outside of their context of use (Dando, 2011, p.9). They also place too much emphasis on the scientific community as custodians of responsibility, and fail to take into account broader institutional, political and societal dimensions of “responsible innovation” that come to the fore from an STS perspective (Stilgoe *et al*, 2013). They also unhelpfully cast scientists as “naive dupes”. As McLeish and Nightingale (2007, p. 1648) point out, “framing dual use in terms of technology transfer, and consequently framing the scientific community as naively transmitting dangerous knowledge and materials, is unlikely to enculture scientists to cooperate”. In interviews with UK scientists, McLeish and Nightingale found that they “were far more willing to become actively engaged with biosecurity governance, and were willing to devote considerable amounts of time to it, if they were seen as ‘guardians' rather than ‘naïve dupes' ” (ibid., p. 1648).

### The encounter between dual use and synthetic biology discourses

In the early 2000s, the discourse on dual use that we have analysed encountered the discourse promulgated at the time by scientists such as Endy, Carlson, Keasling and Church to promote synthetic biology as a radically new emerging field of science. As de-skilling and black boxing of complex knowledge was a central part of the promise of synthetic biology, these two discourses resonated well together, and were mutually reinforced by their encounter. The heightened sensitivity to bioterrorism in the post-9/11 context, and the contemporaneous shift in focus towards dual use research (as opposed to dual use technology) in this period further contributed to this coalescence.

For example, the statement by Mukunda *et al* (2009) that synthetic biology represents “a sustained, well-funded assault on the necessity of tacit knowledge in bioengineering” was directly derived from the discourse of prominent synthetic biologists, in particular Drew Endy.^8^ In his programmatic review of the field, Endy (2005) spoke about the need for an “abstraction hierarchy” whose purpose “is to hide information and manage complexity” and which would enable individuals “to work independently at each level of the hierarchy”. In the cartoon published in *Nature* alongside Endy's review, the heroine scientist states: “The entire point of all this is that we are gonna hide all these details inside a black box so that you don't have to remember all this stuff” (accessible at: openwetware.org/wiki/Adventures).

Endy explicitly made a connection between these goals and potential “new biological threats”. For example, in an early report intended to generate funding from US government agencies, he stated that “the development of technologies for engineering biology appears inevitable, and their distribution uncontrollable” (Endy, 2003). The study involved 34 US researchers, including several who have since become key spokespersons for synthetic biology. They outlined a “roadmap” for what was then a fledgling field and summarised their findings in three bullet points: (i) “Biology is a powerful technology” (ii) “Biological technology poses a danger on par with any past experience” (iii) “Synthetic biology advances science & technology while mitigating danger” (p. 3). Figure 1 is from this report, and was intended to portray how “the same technologies that are needed to help enable rapid responses to new biological threats could also be used to help create the threats themselves” (p. 16).

The way in which founding leaders of synthetic biology participated in constructing the association between synthetic biology and biosecurity threats is also illustrated by these examples: Rob Carlson first published his influential “Carlson curves”, illustrating the increasing productivity and reducing cost of DNA synthesis, in an article published in a biosecurity journal (*Biosecurity and Bioterrorism*) in which he argued that “the proliferation of skills and materials is inevitable” (Carlson, 2003, p. 7); George Church published his *Synthetic Biohazard Non-Proliferation Proposal* in 2004 (Church, 2004); and the *Options for Governance* report commissioned by the JCVI focused almost entirely on biosecurity risks (Garfinkel *et al*, 2007). Thus leading synthetic biologists seemed to be, from the start, strikingly comfortable with talking about the great dangers posed by the work that they were promoting. This can be explained in part by the fact that highlighting those dangers emphasised the power of the technology and a particular vision of the “engineering of biology”. This was useful for attracting political support and funding to the field. It also serves to present themselves as thoughtful, deliberative scientists addressing ethical issues ahead of inevitable criticisms. Moreover, raising these concerns also enabled synthetic biologists to emphasise the field's ability to counter these potential threats.

## Uncomfortable Knowledge for Synthetic Biologists

### The synthetic biology/engineering conundrum

Thus, heightened concerns about the biosecurity threat posed by the potential dual use of synthetic biology are deeply interconnected with the particular way in which these leading synthetic biologists portrayed “de-skilling” and “engineering”. As a result, “de-skilling” has become something that is advocated by synthetic biologists but feared by governmental and inter-governmental actors responsible for managing biosecurity threats. This became very apparent during discussions at our workshop, where we identified a quandary that we characterised as the “synthetic biology/engineering conundrum”:^9^

On the one hand, if tacit knowledge remains important in synthetic biology, then this implies that it will not be easily accessible to outsiders and this reduces concerns about the dual use threat. On the other hand, if synthetic biology is an engineering discipline **and if** this means that we overcome the barriers posed by tacit knowledge, then this implies that it could become more accessible to outsiders and this increases the dual use threat. Thus, biosecurity concerns are heightened when the more extreme depiction of synthetic biology's ability to engineer biology is emphasised. (Jefferson *et al*, 2014b, p. 46) (Emphasis in original)

We also noticed that this conundrum was very apparent to STS scholars participating in the workshop, who commented on it during the day, but was not so obvious to synthetic biologist present. This is evident in this intervention by a synthetic biologist:

what I don't understand about this debate […] is that bioterrorism is an act by a part of society, a human act to do something, to harm someone, whatever. You social science guys will probably say I've got it all wrong, but that's my simplistic understanding of what a bioterrorist is. So the technology is kind of disentangled from that and I don't understand why synthetic biology technology is being entangled with automatically bioterrorism. I just don't get that. You know, are you saying that, as the technology becomes so good there will be an increase in bioterrorism, is that the correlation? So I just don't understand why there's such a close interlink.

The dynamics of these discussions revealed the way in which STS perspectives about tacit knowledge can be experienced as uncomfortable knowledge by synthetic biologists. Broadly speaking, the flow of discussions at the workshop went as follows, and this reflected the dynamics of discussions we have witnessed in many other arenas. First, scholars from the field of STS (including Kathleen Vogel and Sonia Ben Ouagrham-Gormley, who both made presentations at the workshop^10^) emphasised the sociotechnical barriers to the development of bioweapons, including tacit knowledge and organisational dimensions. In response, synthetic biologists emphasised the power of the engineering approach to overcome those barriers and to enable even relative amateurs (such as PhD students, DIY biologists and iGEM teams) to successfully perform genetic engineering easier, faster and cheaper than ever before. For example, responding specifically to Ben Ouagrham-Gormley's talk, one synthetic biologist at the workshop said:

It's good to see that my lab was a microcosm of 60 years of Soviet failure because, in fact, it happens in everybody's lab. You get communication breakdown, you get protocols that are dodgy, all sorts of things, but I think the real point to synthetic biology is that it's engineering. So a lot of these things will get taken away because what we're trying to do is to make it so that we don't have to have a top to bottom design issue. […] so we will have things like a design abstraction hierarchy where you can design at different levels of the process and it will still integrate, and that's clearly something that hasn't happened in the past. […]. So lots of these things will go away because this is engineering, not biology, not just science anymore, and so I think, hopefully, synthetic biology will help us out in that regard and I think [names two other synthetic biologists in the room] certainly wouldn't disagree with that.

This participant also described how the engineering approach of synthetic biology was leading to “bulletproof protocols” for routine experimental procedures such as the production of competent cells. We see here how ignoring – or rather re-interpreting – the important role of tacit knowledge as a fundamental part of all scientific endeavours is part of what sustains a particular vision of synthetic biology, because it supports the claim that the engineering approach of synthetic biology will “take it away”. In order to facilitate this, the problems encountered by the Soviet bioweapons programme, which Vogel and Ben Ouagrham-Gormley attribute to tacit knowledge and other intangible and institutional barriers was re-defined here as being due communication problems, dodgy protocols, and the absence of a design abstraction hierarchy.

The final stage in this flow of discussions was that biosecurity experts at the workshop responded to this portrayal of the “engineering of biology” by expressing their fears that this could be misused by malevolent actors. This illustrates the reverberation between the way in which dual use is understood among actors in the biosecurity field and a particular synthetic biology discourse that has occurred in policy arenas over the last decade or so, and which has led to the presumption that synthetic biology is likely to significantly increase the risk of attacks from bioweapons.

### Re-interpretation and re-deployments of “de-skilling”

At this point, however, something happened at our workshop that we have not routinely encountered in more public arenas. Reacting to the fears expressed by biosecurity experts, some of the synthetic biologists present provided a different interpretation of “de-skilling”, as illustrated by this intervention:

So you were asking what happens if we consider [synthetic biology] as an engineering discipline and what are the consequences in terms of the risks that it implies? I will draw a parallel that is worth what it's worth. If we take a plane which is built of quite a large number of well-characterised parts in a very methodic and systematic way, how many people on the street can build a plane, make it fly and basically use it as we use it today for commercial transportation? Not so many. It's the same thing. Deskilling doesn't mean that you don't need a large infrastructure and a team effort to get things to work properly and I think that this is at the core of the debate here. When we say deskilling or when we say making the engineering of biology easier, that doesn't mean that you don't require a team effort and a very solid infrastructure to support actually the development of this, what we call deskilling or engineering of biology. So addressing the question in a simplistic way saying, okay, if we have tacit knowledge then it's not so dangerous, and if we have engineering of biology as we claim we will have in synthetic biology then it becomes much more dangerous de facto is not true just for that reason.

Another synthetic biologist stated:

the point is in my view we should not be interpreting deskilling in let's say the obvious way. What we actually mean by deskilling is the whole process becomes much more systematic. The underlying theme here is what happened in the Industrial Revolution, that the deskilling process was one of moving from a few highly skilled craftsmen to making a systematic process. That's not to say there isn't a lot of skill in all these industrial processes, but that's what we mean by deskilling.

This portrayal of “de-skilling” and of the “engineering of biology” is different from the one deployed earlier in the discussion, because specialised expertise, teamwork, complicated machinery, trouble shooting and thus a plethora of organisational dimensions would continue to be required when synthetic biology's goal to engineer biology succeeds. Comments received from workshop participants on a draft of the report further illustrated the way in which this interpretation of de-skilling was mobilised in the context of debates about the dual use threat posed by synthetic biology. In response to our description of the conundrum, one synthetic biologist commented “I simply do not believe that it is easier to produce bioweapons as a result of synthetic biology. This is too simplistic a statement”. They also stated: “[i]n most of the examples I can think of, de-skilling does not make things easier it makes them more complicated and difficult”. This synthetic biologist accused us of misunderstanding the correct meaning of de-skilling, but these complaints were based on the presumption that we were ourselves making these claims and exaggerating the biosecurity threat, rather than simply reflecting the way in which some participants, and many leading synthetic biologists, have portrayed de-skilling in public arenas.

In this redeployment of the notion of de-skilling, the importance of tacit knowledge and of organisational factors as barriers to “making biology easier to engineer” were now acknowledged, and even portrayed as obvious known knowns that we had not understood. We see here how the same knowns that are denied or dismissed as unknown knowns in certain circumstances can be mobilised as known knowns by the same category of actors in a different context. In this case, unknown knowns were deployed as known knowns when synthetic biologists sought to minimise the dual use threat associated with their field. This strategic move was, however, apparently unconscious and those involved did not acknowledge that this interpretation of de-skilling was very different to the one deployed by founding leaders of synthetic biology.

These different interpretations of de-skilling reflect, to some extent, differences in the vision of US synthetic biologists such as Endy and Carlson on the one hand (who were not present at our workshop), and of the UK synthetic biologists participating in our workshop. However, Endy's vision of the engineering of biology was also very much present at the workshop, as illustrated by the earlier quote from a synthetic biologist. Moreover, the last sentence of that quote demonstrates that this participant assumed that there was agreement among synthetic biologists in the room around this portrayal of the engineering of biology as “taking away” the barriers described by Vogel and Ben Ouagrham-Gormley.

The dynamics observed at our workshop demonstrate that knowledge that is uncomfortable when promoting the revolutionary nature of the field (in this case the prevalence and importance of tacit knowledge and institutional dimensions in technoscience) can become comfortable – and even necessary – to sustain the institutional goals of synthetic biology when the association with an increased biosecurity risk was experienced as a threat. Moreover, we have observed instances where knowledge about tacit knowledge is mobilised by synthetic biologists as a known known to promote the revolutionary nature of the field. Thus, we have routinely witnessed situations where synthetic biologists acknowledged – or even stressed – the importance of tacit knowledge in *previous* forms of genetic modification research (for example, molecular biology), in order to highlight the power of the engineering approach of synthetic biology to eradicate it.^11^ This happened during our workshop, as illustrated by this intervention:

The whole point of synthetic biology is to take the pain out of molecular biology, because it's a painful process. I've got the scars, but future synthetic biologists will have no scars, they'll have beautiful skin, all of that pain will have gone.

We have also observed instances where the importance of tacit knowledge and other intangible barriers was simply dismissed. For example, on the day we published our workshop report (21 May 2014), we had the following Twitter conversation with Drew Endy. Claire Marris advertised the online publication by tweeting “synbio becomes scary when its ability to engineer biology is misrepresented”. Less than an hour later, Endy tweeted: “If the judging requirements changed what fraction of iGEM teams could reconstruct a viral pathogen this summer?” and the conversation continued as follows:^12^

Marris: #synbio becomes scary when its ability to engineer biology is misrepresented. Endy: If the judging requirements changed (don't change) what fraction of #iGEM teams could reconstruct a viral pathogen this summer? Marris: Great question Drew! At the heart of the debate. I believe evidence from our workshop suggests the answer is zero. Endy: Unclear from report if your workshop's set of evidence acknowledged specific technical advances over past decade. Endy: eg, engineering basic parts now so reliable, expression failures below 10 per cent; fab costs 10x less; 0% iGEM wishful [Several more tweets here focused on the distinction between making pathogens versus making weapons] Endy: 0% patho. construction wishful thinking. public conflation of pathogen/weapon = trouble. Endy: Which is why I asked you to imagine what would happen if #iGEM rules were different. Do you really believe 0%? Marris: Yes I do e.g. if you can't make quality HeLa cell extract you can't make virus iGEM unlikely to have tacit knowledge Endy: Understood. We disagree then.

Endy's need to defend his own initial interpretation of de-skilling therefore seems to be stronger than for the UK synthetic biologists cited above. Campos (2013, p. 344) explains how Endy switched, around 2010, “from a more revolutionary rhetoric to a strategic downplaying of the novelty and risk of the new field when faced with powerbrokers in higher circles for whom such qualities might have raised concerns” and how this occurred at the time when Endy transitioned “from the outsider hacker” to “finally becoming a voice of transposed authority for the entire field”. This supports Rayner's assertion that institutions need to develop and then sustain simplified, self-consistent worldviews in order to act, but reveals that these worldviews can evolve over time. Our analysis suggests, however, that the kind of transition in rhetoric described by Campos does not only take place over time, but also across “spaces”, and that worldviews are not always self-consistent: Endy, like other actors, is likely to use different rhetoric in different contexts. This resonates with previous work by Marris on the controversy about genetically modified crops (Bonneuil *et al*, 2008). In that analysis, public debate was conceptualised as a set of interactions in diverse arenas. In each arena, particular forms of argumentation and action carry most weight, which means that different framings of an issue can co-exist across arenas. However, when public controversies develop, and the stakes increase, interactions between arenas intensify and these different framings confront each other. The competing interpretations of the notion of “de-skilling” and “engineering” revealed in our analysis may illustrate the emergence of this phenomenon in debates about the risks associated with synthetic biology.

## Uncomfortable Knowledge for Biosecurity Experts

The same knowledge that is uncomfortable for synthetic biology institutions is also uncomfortable for biosecurity institutions. This means that organisations in both spheres implement strategies to deny this knowledge, and these strategies then bolster each other. For institutions involved in the characterisation and reduction of threats to biosecurity, this knowledge is “disruptive” because it can be perceived as undermining the effectiveness and value of their endeavours. For example, biosecurity experts present at the workshop stressed that “even if the threats associated with synthetic biology are exaggerated, this does not mean they should not be investigated” and “that in a policy context, speculative thinking can be helpful to identify worst case scenarios and potential responses to these” (Jefferson *et al*, 2014a, pages 21 and 43). We suggest that these experts felt the need to highlight these points in order to ward off what was, for their institutions, uncomfortable knowledge.

Biosecurity organisations have become established to manage the threat of bioweapons, largely based on a particular understanding of dual use that is underpinned by the linear model of innovation, does not acknowledge the importance of tacit knowledge (as defined by Collins and Polyani), and ignores the importance of context in determining “dangerousness”. Denying or dismissing the three unknown knowns we identified is therefore necessary to sustain their existence. Developing foresight scenarios based on speculative assumptions is an example of the type of activities performed by these organisations, and our suggestion that these help to construct “myths” was not received comfortably by at least one security expert who participated in our workshop:

the term, ‘myths' […] I certainly found stimulating but I think perhaps it's drawing our attention away from certain interesting matters that are particularly relevant in the context of the BWC and people who are interested in more long-term views on how our existing regulations will respond to challenges. I think misuse scenarios or hypotheses they draw on serve a variety of roles in different contexts. In a policy context such myths are used to pose questions to existing regulatory systems. […] The last thing you want, really, is issues to be dealt with on the hoof […] I think sometimes speculative discussions can encourage responsible conversation and can be useful.

More generally, any knowledge claim that reduces perceptions of dual use risks can be interpreted as threatening by actors from biosecurity organisations because, as stated jokingly by Alexander Garza (assistant secretary for health affairs and chief medical officer of the US Department of Homeland Security) during the PCSBI meeting discussed earlier, they are “paid to be paranoid” (PCSBI, 2010b, Session 6).

## Discussion

### The synthetic biology/engineering conundrum resolved

We identified three bodies of knowledge from the field of STS that are relevant to understandings of the dual use threat posed by synthetic biology and showed how they have been systematically ignored by institutions involved in the promotion of synthetic biology as well as by those involved in managing biosecurity risks. We claim that this has led to a situation where biosecurity concerns related to the potential misuse of synthetic biology are generally exaggerated. More importantly, we argue that these concerns are *misplaced* and that the particular worldview that is sustained by this structured ignorance directs the policy gaze towards measures that, on their own, have limited effects on security.

The fact that the unknown knowns identified are experienced as uncomfortable knowledge by these institutions is intriguing, because acknowledging them could be very comforting, in the sense that it would mean that we should not worry so much about the potential misuse of synthetic biology and other biosciences by bioterrorists. The seemingly counterproductive promotion of the biosecurity “perils” associated with synthetic biology by some of its most fervent advocates can however be understood when one recognises how it serves to sustain a particular vision of the “promises” of the field, which are both founded on particular portrayals of “de-skilling” and the “engineering of biology”.

### The reverse argument

We believe that this phenomenon means that policies aimed at *promoting* the field of synthetic biology are also often misdirected. As we have shown, the simplistic and discredited linear model of innovation that underscores the dominant understanding of the dual use threat posed by advances in the biosciences leads to an over-estimation of the smoothness and ease of innovation for biological weapons development. It also leads to an *under*-estimation, and misunderstanding, of the challenges involved in facilitating the development of synthetic biology for *beneficial* purposes. The sociotechnical dimensions that constitute barriers to the delivery of the promised perils of the field are also barriers to the delivery of the promised benefits of the field. This means that revolutionary developments in science do not necessarily make it easier to produce technology. Improvements in scientific methods may be relevant to certain stages in the production of technologies, but those may not be the rate limiting step. This is true for bioweapons *and* for commercial biotechnology.^13^ This helps explain why insights about the specific, local, sociotechnical barriers revealed by STS and innovation studies is uncomfortable for institutions that promote “basic” scientific research on the basis that it will necessarily “translate” into beneficial technologies. Acknowledging this disruptive knowledge could lead to more effective strategies for the commercial development of biotechnologies derived from synthetic biology.

### Clumsy solutions

This article has shown how actors who share a particular institutional culture will strategically construct knowledge and ignorance in ways that are most compatible with the goals of that institution. This is necessary for those institutions to function, but we have shown how, in this dysfunctional case, this phenomenon has led to erroneous threat assessments and misguided policies. Rayner suggests “that ‘clumsy' arrangements may need to be constructed to ensure that uncomfortable knowledge is not excluded from policy debates” (op cit., p. 107). These “clumsy solutions” would involve bringing together “multiple, diverse, perhaps incompatible, perspectives” and would result “in a settlement that is inelegant from any single perspective, but robust because it relies on more than one epistemological and ethical foundation” (p. 123).

The workshop we organised to bring together synthetic biologists, security experts and social scientists was an attempt at such a clumsy solution. Bringing together this diverse group of actors in a relatively closed and safe space^14^ revealed tensions between different epistemological perspectives, and between different understandings of synthetic biology, bioterrorism and dual use. In the report from that workshop, we suggested that acknowledging the kinds of dynamics discussed in this article could help to develop more productive discussions (Jefferson *et al*, 2014b, pp. 46, 48). However, as Rayner (2012) notes, making competing interpretations explicit can “cause delicate institutional arrangements to fracture” (p. 113). Thus, the key is to construct “arrangements which permit different sub-sections of a society or organization to rub along with each other by not questioning each other's motivations and worldviews too deeply” (Rayner, 2012, p.112). Drawing on their own experience of transdisciplinary collaborations in neuroscience, Des Fitzgerald, Melissa Littefiled and their colleagues have discussed how this can lead, in transdisciplinary research collaborations, to “subterranean logics of ambivalence, reserve and critique” (Fitzgerald *et al*, 2014, p. 2). They have also argued that participants in such collaboration experience “disciplinary double consciousness” and may need to experience this as “a useful collaborative position and tool” (Littlefield *et al*, 2014, p. 2). From our experience of working towards clumsy solutions through transdisciplinary collaborations in synthetic biology, this involves a difficult balancing act between on the one hand sustaining fragile institutional arrangements, and on the other hand making competing epistemological perspectives more explicit and discussable. This can be very uncomfortable indeed, but we remain committed to the idea that it is a worthwhile endeavour.

## Figures and Tables

**Table 1:** Misdirect policy gaze in discussions about the dual use threat of synthetic biology

*Policy discussions tend to focus on*	*Policy discussions do not tend to focus on*
How to make *pathogens*	How to make biological *weapons*, especially weapons of *mass destruction*
	
*Biosecurity* risk from deliberate release of pathogens	*Biosafety risk* from unintended release of pathogens
	
*Discrete pieces of research*	*Innovation regimes and processes*
	
*Codified knowledge* (for example, publication of “experiments of concern”)	*Tacit, local and collective nature of knowledge* (learning by doing, team work, troubleshooting)
	
*Tangible materials* (synthetic DNA, pathogens, “select agents” and hardware such as fermenters, incubators)	*Intangible barriers* (macro- and micro-level organisational dimensions, infrastructure)
	
*Rogue outsiders*	*Legitimate insiders*
*Amateurs*	*Professionals*
*Non-state actors* (bioterrorists, DIY biologists)	*State-sponsored activities* (military biodefense programmes, civil biopreparedness programmes )
	
*Ethics and responsibility of individual scientists*	*Institutional and political dimensions of responsible innovation*

## Five “myths” that underlie policy debates on synthetic biology and biosecurity

*Myth *1: Synthetic biology is de-skilling biology and making it easier for terrorists to exploit advances in the biosciences;
*Myth *2: Synthetic biology has led to the growth of a DIYbio community, which could offer dual use knowledge, tools and equipment for bioterrorists seeking to do harm;
*Myth *3: DNA synthesis has become cheaper and can be out-sourced, and this will make it easier for terrorists to create biological threat agents;
*Myth *4: Synthetic biology could be used to design radically new pathogens;
*Myth *5: Terrorists want to pursue biological weapons for high consequence, mass casualty attacks.

*Source*: Jefferson *et al* (2014a).

## Footnotes

1 Other relevant literature on the social construction of ignorance includes Balmer (2006, 2012) on “secrecy and science”, who was one of the first to urge researchers to “take space and knowledge seriously” (2006, p. 75); Hilgartner (2012) on the “selective flow of knowledge” and Milne (2012) on the “geography of science” (see also Lentzos, 2014).

2 A list of some of our higher profile engagements can be found here: www.kcl.ac.uk/sspp/departments/sshm/research/Research-Labs/CSynBI@KCL-Impact.aspx

3 We indicate here (and elsewhere throughout this article), where possible, the number of times documents have been cited according to the Google Scholar database (accessed 25 July 2014), in order to emphasise how particular publications have become touchstones in these debates.

4 As we discuss later, Mukunda *et al* are simply replicating here the discourse of prominent leading founders of the field of synthetic biology.

5 Even after publication of Tucker's 2011 article, citations to the 2006 article predominate (28 out of the 96 citations to the 2006 article are from the years 2012–2014).

6 The influence of Tucker and Zilinskas's 2006 article is also because of the title, “The Promises and Perils of Synthetic Biology”, and thus the fact that it has become a touchstone illustrates the power of the “promises and perils” framing of debates about synthetic biology (Jefferson *et al*, 2014b, p. 16–18).

7 We (Revill and Jefferson, 2014; Jefferson *et al*, 2014a) and others (Grushkin *et al*, 2013) have showed that this focus is incommensurate when compared with the low level of sophistication of the research activities conducted by DIY biologists, and their willingness to be open and transparent about their activities.

8 For an excellent account of the role played by Drew Endy in establishing a particular vision for the field of synthetic biology, see Campos (2013).

9 This quandary was also discussed by Tucker in his 2011 article (Tucker, 2011, p. 69).

10 Summaries of all the workshop presentations are given in the report (Jefferson *et al*, 2014b) and the slides used can be accessed here: www.kcl.ac.uk/sspp/departments/sshm/research/Research-Labs/CSynBI-Events.aspx

11 This is also apparent in the earlier citation on tacit knowledge from Mukunda *et al* (2009) and the quote from the workshop participant that begins “It's good to see that my lab was…”..

12 This is an edited extract from a longer Twitter conversation on 21 and 22 May 2014. The full transcript is accessible here: https://www.academia.edu/8220916/Twitter_conversation_with_Drew_ Endy_May_2014

13 We thank Paul Nightingale for helping us to articulate this point more clearly.

14 The workshop was by invitation only and was held under the Chatham House rule.
